# Strategies to manage anterior disc displacement without reduction of temporomandibular joint: a case report

**DOI:** 10.1080/07853890.2021.1896838

**Published:** 2021-09-28

**Authors:** F. Ricardo, P. Moleirinho-Alves, P. Cebola, E. Januzzi, A. M. Almeida

**Affiliations:** a Clínica Cuf Alvalade, Lisboa, Portugal; b Private Clinic Pratice; c Escola Superior de Saúde Egas Moniz (ESSEM), Egas Moniz Cooperativa de Ensino Superior, Caparica, Portugal; d Centro de Investigação Interdisciplinar Egas Moniz (CiiEM), Almada, Portugal; e Instituto Universitário Egas Moniz (IUEM), Egas Moniz Cooperativa de Ensino Superior, Caparica, Portugal

## Abstract

**Introduction:**

Articular disc displacement is the most common temporomandibular joint (TMJ) arthropathy [1]. Disc displacement without reduction (DDwoR) is an intracapsular biomechanical disorder involving the condyle-disc complex [1] and can cause TMJ pain, limited mouth opening [2] and a change in the opening pattern of the patient. At first, the treatment for DDwoR should be reversible and conservative and include drugs, interocclusal devices and physiotherapy (PT) [3]. Moreover, minimally invasive techniques such as viscosupplementation (VS) seems to reduce pain and symptoms associated with internal derangement and improve quality of life [4]. The aim of this study is to report the efficacy of a combined protocol (PT and VS) in the control of signs and symptoms of a patient with temporomandibular disorder (TMD).

**Materials and methods:**

Female patient, 38 years, with signs and symptoms of TMD presents a clinical diagnosis of right TMJ DDwoR and left TMJ subluxation, according with the Diagnostic Criteria for Temporomandibular Disorders protocol (DC/TMD). The initial pain free opening (PFO) was 31 mm with an uncorrected deviation to the right side in terms of opening pattern. Interdisciplinary team composed by two dentists and two physiotherapists decided the following strategy: assessment of PFO and opening pattern (DC/TMD examination form) before and after any intervention. After the initial assessment we had the first session of PT to prepare for the VS composed of behavioural and cognitive therapy, manual therapy, motor control exercises and prescription of home exercises. One week after we performed the first session of VS with 1 ml of high molecular weight hyaluronic acid bilaterally in the TMJ followed by another session of PT. Two sessions were performed, once a week, in two consecutive weeks. All the assumptions of the Helsinki Declaration have been fulfilled and an informed consent for clinical case of Clínica Dentária Egas Moniz approved by the ethic commission of Instituto Universitário Egas Moniz.

**Results:**

Data was collected before and after any intervention. In first session of PT, PFO measurements increased from 31 mm to 38 mm and opening pattern remained a right uncorrected deviation. After the VS and second PT session the PFO increased from 35 mm at the beginning of the intervention to 45 mm at the end and the opening pattern changed to a straight opening pattern.

**Discussion and conclusions:**

VS has been proven to be effective for knee and other large joints [4]. Studies suggest that adding VS to PT and therapeutic exercise may increase functionality [5]. No significant differences between non-invasive conservative interventions and minimally invasive or invasive surgical interventions for TMJ DDwoR [2] so primary treatment should consist of conservative strategies [6] carried out by a multidisciplinary team in order to improve range of motion, restore biomechanics, dynamic lubrification and function. This case report suggest the effectiveness of the combined protocol (PT and VS) in the control of signs and symptoms of TMD reinforcing the role of the interdisciplinary team. However, further studies should be done with an increased sample.
